# A comprehensive national survey on malocclusion prevalence among Palestinian children

**DOI:** 10.1186/s12903-024-04432-1

**Published:** 2024-06-07

**Authors:** Hamsa Amro, Shahenaz Najjar, Majdi Owda, Firas Elayyan

**Affiliations:** 1 Preventive Department, Oral and Dental Health Unit, Ministry of Health, Ramallah, Palestine; 2 Health Sciences Department, Faculty of Graduate Studies, Arab American University, Ramallah, Palestine; 3 Leuven Institute for Healthcare Policy, Pillar Quality and Safety, Department of Public Health and Primary Care, KU Leuven, Belgium; 4 UBI Business School, Brussels, Belgium; 5 WHO Regional Office for Europe, Europe, Denmark; 6 Faculty of Data Science, Arab American University, Ramallah, Palestine; 7 Private clinic, Ramallah, Palestine

**Keywords:** Malocclusion, Orthodontic, Prevalence, Surveillance, Schoolchildren, Palestine

## Abstract

**Background:**

This study aims to evaluate the prevalence of malocclusion and orthodontic features among schoolchildren in the West Bank, Palestine.

**Methods:**

A stratified cluster sample of 1278 schoolchildren (620 males, 658 females, mean age 12 years and 5 months (± 0.5)) were examined. Candidates who had not received any previous orthodontic treatment were only included. Dental anomalies like missing and ectopic teeth were recorded. The anteroposterior occlusal relationship was assessed based on Angle classification. Overjet and overbite were measured. Crowding and spacing were recorded subjectively. In addition, crossbite, openbite, and midline displacement were recorded. The chi-square test and descriptive analysis were used statistically.

**Results:**

The study found Angle Class I molar relationship in 65%, Class II div 1 in 17%, Class II div 2 in 6%, and Class III in 12% of the sample. An overjet (OJ) of more than 4 mm was present in 17%, and 4% had OJ of more than 6 mm; an OJ of at least 0 mm or less in 36%, and 6% had a reverse OJ. A normal overbite was observed in 53%, while 28% had an increase and 19% had a decreased overbite. An anterior openbite (AOB) was present in 9%, and a scissor bite or anterior crossbite in 6% and 14%, respectively. A posterior crossbite was observed in 12% (9% unilateral and 3% bilateral). Midline displacement was found in (9%). Crowding was observed in 35% and 31% and spacing in 24% and 15% of the maxillary and mandibular arches, respectively. A statistically significant relationship between gender and midline shift, a diastema, spacing in the upper arch, and most dental anomalies was found; males were more affected (*p* < 0.05).

**Conclusion:**

This study reported a high prevalence of malocclusion among schoolchildren in Palestine. A collaborative effort should be directed to obtain more monitoring and surveillance of malocclusion more frequently to prevent and control the exacerbation of the problem.

## Introduction

Malocclusion and subsequent dental discrepancies are considered one of the most frequently reported problems among children and adolescents [1]. Unlike other oral health conditions, malocclusion rarely causes severe pain. It is associated with low quality of life, low self-esteem, and social, psychological and functional disturbances [2]. Many researchers found a significant relationship between malocclusion, poor oral function, poor aesthetic appearance, speech difficulties, periodontal disease, dental caries, Temporomandibular joint disorders, and unfavourable psychological disorders [2].

Most studies measuring the prevalence of malocclusion and related factors depend on epidemiological investigations as a method of choice. The prevalence of malocclusion varies between different countries and geographic regions. The main objective of measuring the occlusal traits is to determine the extent of the health problem and provide the necessary data for setting priorities and developing health policies. Numerous studies have been conducted on the prevalence of malocclusion and the need for treatment worldwide using several indices. Borzabadi-Farahani et al., 2009, studied the prevalence of malocclusion and occlusal traits in 502 Iranian adolescents and found an increased overbite in 34.5%, a severe crowding in the maxillary arch in 16.7%, an angle Class II division 1 in 24.1% [3]. Alkateeb et al., 2005, in a cross-sectional study, examined occlusal traits depending on Bjork et al. methodology and reported an overall prevalence of malocclusion of 92% among Jordanian children aged 13 to 15, an Angle Class II and III malocclusion in 18.8% and 1.4%, crowding in 50.4% and midline shift in 31.7% of their sample [4]. With regard to the considerable variation in the reported prevalence of malocclusions and the minimal data available, particularly in adolescents, the present study aimed to establish the frequencies of the different occlusal traits among adolescents in the West Bank of Palestine.

## Materials and methods

### Study population

The study was approved by the local ethics committee (Palestinian Health Research Council) (PHRC/HC/998/21). The target population for the present cross-sectional study consisted of 7th-grade Palestinian public-school children in West Bank, Palestine. To meet national needs and international requests, a Palestinian oral health surveillance programme screen public schoolchildren in the 1st (6-year-olds), 7th (12-year-olds), and 10th (16-year-olds) grades. Only data for 7th grade was collected. West Bank has 17 governorates, according to data from the Ministry of Education’s 2019 statistical book. Around 53,247 seventh-grade students were distributed among 1070 schools (454 northern, 254 central, and 362 southern public schools), with 408 female schools, 386 male schools, and 276 mixed schools.

### Sample size and clinical examination

To ensure random selection, 69 public schools were chosen from different geographic locations in the West Bank using a stratified selection technique. The sample size was calculated prior to data collection using the expected sample size formula [5]. Assuming the prevalence of malocclusion to be 0.50 in children with and without orthodontic anomaly, a significance level of 5%, power of 95% and a design factor of 1.2 (increasing the sample size by 20%), The result of the equation is multiplied by three domains (northern, central and southern regions of West Bank). The calculated sample was 1384.

The selection criteria for examination included children enrolled in the seventh grade and within the defined age range of 12.0 to 12.9, without any systemic or local health conditions, such as cleft lip and palate, or other syndromes. Exclusion criteria for this study comprised children with a history of orthodontic treatment and/or those who had undergone extraction of any first molars. Following the application of both inclusionary and exclusionary criteria, the final eligible sample comprised 1278 children.

The children were examined at school, under room light, with a mouth mirror, tongue depressor, sharp pencil and triangle set square ruler (for measuring overjet and overbite). For every child, a registration chart related to malocclusion was established which included all variables [6–11]. The following parameters and criteria were used in the present study.

#### Sagittal dimension

Assessment of the anteroposterior molar relationship was based on Angle’s classification. Overjet (OJ) was measured in mm by measuring the distance between the most prominent point on the incisal edges of upper and lower central incisors, considering that the normal value ranged from 2 to 4 mm, increased if it is more than 4 mm and decreased if it is less than 2 mm, and reversed overjet if it is less than 0 [12–14]. OJ was recorded as follows: < 0, 0 ≤ to < 2, 2 ≤ to ≤ 4, 4 < to ≤ 6, and > 6 mm. An anterior crossbite (centrals and laterals) also included subjects with a reverse OJ.

#### Vertical dimension

The overbite was considered normal if values ranged from 2 to 4 mm, increased if it was more than 4 mm and decreased if it was less than 2 mm [12–14]. Anterior openbite relationship was noted if all four upper incisors do not overlap any lower incisor in centric occlusion. At the same time, a posterior openbite was recorded when there was a lack of contact between the posterior teeth (premolars and 1st molar).

#### Transverse dimension

A posterior crossbite was registered when the maxillary premolars and/or molars’ buccal cusps occluded lingual to the mandibular antagonists’ buccal cusps (uni- or bilateral). Teeth that were edge-to-edge were also included. A scissor bite was registered when any of the maxillary premolars or molars occluded with the buccal surface of the mandibular antagonists. Midline shift was diagnosed when the midlines of the maxillary and the mandibular arch are displaced by 2 mm or more regardless of the displacement occurs in the maxillary or mandibular arch.

#### Alignment anomalies

The presence of crowding, spacing, or diastema was recorded subjectively according to the overlapping of erupted teeth or lack of space over 2 mm for space discrepancies, while diastema was diagnosed when there was a space of at least 2 mm between the maxillary central incisors [6, 15].

#### Dental anomalies

Anomalies in the dentition were measured by recording the number of missing permanent, ectopic, supernumerary, malformed, and retained deciduous teeth.

No radiograph of any kind was used.

### Statistical analysis

Data processing and statistical analysis were undertaken using Statistical Packages for Social Science (SPSS Inc., Chicago, Illinois, USA) version 23.0. The chi-square and Fisher’s exact tests examined sexual dimorphism (Angle Classes, OJ, overbite, crossbite, scissor bite, midline discrepancy, and crowding/spacing). A 2-sided α of < 0.05 was considered statistically significant. Inter-examiner reliability was determined by 14 observers evaluating 32 children on the same day independently from each other. The intraclass correlation coefficient (ICC) and its corresponding 95 per cent CI were calculated for the occlusal data. Inter-examiner reliability was tested and found consistent for all measurements.

## Results

A total of 1278 children met the inclusion criteria. Of these, 620 were males (48.5%) and 658 were females (51.5%), mean age 12.5 (± 0.5).

### Sagittal dimension

According to Angle classification, Class I molar occlusion was found in 65% of children, while Class II and Class III were recorded in 23% and 12%, respectively. OJ of more than 4 mm was present in 17%, and 4% had OJ of more than 6 mm; an OJ of at least 0 mm or less in 36%, and 6% had a reverse OJ. An anterior crossbite was found in 14%. There were no significant differences in diagnosis between males and females (*p* > 0.05) (Tables 1 and 2).

**Table 1:** Prevalence of molar relationship according to Angle classification

		Male	Female	Total
		n (%)	n (%)	n (%)
Molar relationship	Class I	387 (63%)	435 (66%)	822 (65%)
	Class II div I	102 (17%)	108 (16%)	210 (17%)
	Class II div 2	42 (7%)	39 (6%)	81 (6%)
	Class III	84 (14%)	73 (11%)	157 (12%)

* P-value > 0.05, ** P-value > 0.001 (used χ² person and Fisher Test)

### Vertical dimension

The prevalence of deep overbite (> 4 mm) was 28% and overbite (2 ≤ to ≤ 4 mm) accounted for the majority. AOB was present in 9% (Table 2).

### Transverse dimension

Of the transverse dimension, a midline shift (≥ 2 mm) was recorded in 29%. Males experienced more midline shift (32%) than females (29%, *p* < 0.05). A unilateral posterior crossbite was found in 9%, while a scissor bite was registered in 6% (Table 2).

**Table 2:** Prevalence of sagittal, vertical, and transverse occlusal anomalies

		Male		Female	Total
		*n* (%)		*n* (%)	*n* (%)
Sagittal					
Overjet	< 0 mm	45 (7%)	32 (5%)		77 (6%)
	0 ≤ to < 2 mm	180 (29%)	210 (32%)		390 (30%)
	2 ≤ to ≤ 4 mm	285 (46%)	315 (48%)		600 (47%)
	4 < to ≤ 6 mm	85 (14%)	79 (12%)		164 (13%)
	> 6 mm	25 (4%)	22 (3%)		47 (4%)
Anterior crossbite		88 (14%)	89 (14%)		177 (14%)
Vertical					
Overbite	< 2 mm	115 (19%)	127 (19%)		242 (19%)
	2 ≤ to ≤ 4 mm	328 (53%)	347 (53%)		675 (53%)
	> 4 mm	177 (28%)	183 (28%)		360 (28%)
Anterior openbite		61 (10%)	52 (8%)		113 (9%)
Posterior openbite		109 (18%)	91 (14%)		200 (16%)
Transverse					
Posterior crossbite	unilateral	59 (59%)	55 (8%)		114 (9%)
	bilateral	26 (4%)	11 (2%)		37 (3%)
Scissor bite		36 (6%)	38 (6%)		74 (6%)
Midline shift		196 (32%*)	169 (26%)		365 (29%)

* P-value > 0.05, ** P-value > 0.001 (used χ² person and Fisher Test)

### Alignment and dental anomalies

Alignment and dental anomalies were presented in Tables 3 and 4; the prevalence of crowding of the maxillary or mandibular teeth was 35% and 31%, respectively. 20% of children had both upper and lower teeth crowding. The rate of spacing of the maxillary or mandibular teeth was 24% and 15%, respectively. 9% of the children had upper and lower teeth spacing. Spacing in the maxilla was significantly higher in males than in females (*p* < 0.05). The prevalence of dental anomalies is reported as background information related to malocclusion classifications. Retained deciduous teeth were found in 30% of children, Ectopic eruption in 19%, and permanent teeth missing due to extraction, trauma, or congenitally in 15%, while malformation and supernumerary were rare. Essential sex differences were observed in ectopic eruption and missing permanent teeth.

**Table 3:** Prevalence of space anomalies

	Male	Female	Total
	*n* (%)	*n* (%)	*n* (%)
Crowding Upper	206 (33%)	244 (37%)	450 (35%)
Crowding lower	195 (31%)	203 (31%)	398 (31%)
Crowding in both arches	121 (20%)	137 (21%)	258 (20%)
Spacing Upper	169 (27%*)	140 (21%)	309 (24%)
Spacing Lower	99 (16%)	92 (14%)	191 (15%)
Spacing in both arches	55 (9%)	54 (8%)	109 (9%)
Diastema	88 (14%*)	68 (10%)	156 (12%)

* P-value > 0.05, ** P-value > 0.001 (used χ² person and Fisher Test)

**Table 4:** Prevalence of dental anomalies

	Male	Female	Total
	*n* (%)	*n* (%)	*n* (%)
Formation			
Missing permanent teeth	111(18%*)	81 (12%)	192 (15%)
Supernumerary teeth	0 (0%)	1 (0%)	1 (0%)
Malformation	28 (5%)	26 (4%)	54 (4%)
Eruption			
Ectopic teeth	100 (16%8)	137 (21%)	237 (19%)
Retained deciduous teeth	212 (34%*)	167 (25%)	379 (30%)

* P-value > 0.05, ** P-value > 0.001 (used χ² person and Fisher Test

*no radiograph is used

The distribution of missing permanent teeth in the participants’ upper and lower dental arches can be followed in Table 5.

**Table 5:** Distribution of missing permanent teeth

	Upper Arch	Lower Arch
Tooth	n (%)	n (%)
Second Premolar	62 (4.80%)	105 (8.30%)
First Premolar	37 (2.90%)	27 (2.20%)
Canine	66 (5.20%)	26 (2.10%)
Lateral Incisor	24 (1.90%)	4 (0.40%)
Central Incisor	4 (0.30%)	0 (0.00%)

*n is the number of missing teeth occurrence in the defined category, % is the percentage of cases in the defined category

*no radiograph is used

## Discussion

Malocclusion is a handicapping dentofacial anomaly that affects function and esthetic. It affects the quality of life [16]. Studies assessing malocclusion in children have shown inconsistent results. The divergence in prevalence figures may be due to the different methods of registration, inadequate sample size or variations in sample selection, malocclusion indices used, ethnic origin, the era of research, and the stage of dental development [17].

In this cross-sectional study, a sample of 7th-grade school children was selected to obtain preliminary information on the prevalence of malocclusion in the Palestinian population. Our results show that the prevalence of malocclusion is high among schoolchildren in the West Bank in Palestine. This finding corroborates other studies of the same age group in the Middle East [4, 18, 19]. The molar relationship was classified according to Angle classification, which provides a simple way to classify malocclusions. Our findings show that the Class I molar relationship was the most prevalent in this sample, at 65%. This study confirms that Class I is Palestinian schoolchildren’s predominant sagittal molar relationship. The prevalence of Class II malocclusion was 23% (17% Division 1 and 6% Division 2). The prevalence of Class II malocclusion was higher than in Jordanians [4], Indians [20], and East Africans [21]. Palestinian children also showed the exact prevalence of Class II malocclusion as Colombians [22], Iranians [3], Omanis [19], and Lebanese [23].

The prevalence of Class III malocclusion in this study was 12%. This was in accordance with the previous investigations in Iran [24], but it is higher than the rate of 4.3% reported by Elghoul for the Gaza Strip in Palestine [25]. This difference could be because Elghoul used the summer index, which depends on other criteria. The reported prevalence of subjects with a Class III malocclusion in Saudi Arabia [13], Oman [19], Iran [3], and Turkey [26] was comparable with the present result. Palestinian children also showed a lower prevalence of Class III malocclusions compared with Chinese [27].

An OJ value of at least 0 mm or less was present in 36%, 47% had OJ values between 2 and 4 mm, 17% had an OJ of more than 4 mm, and 4% had an OJ of more than 6 mm. The prevalence of an increased overjet of more than 6 mm was consistent with previous studies in Jordanian and Iranian children [3, 4], but less than that observed in Morocco children ^28^, and more than that observed in Brazilian children [28]. This difference could be due to the clinical material used. A reverse OJ (negative) was found in 6% of the studied subjects. It was higher than in Jordanian and Iranian children [4, 24].

The prevalence of anterior and posterior crossbites in the present study was 14% and 12% (9% unilateral, 3% bilateral), respectively. Farahani et al., 2009, in a study of malocclusions in Isfahan, Iran, reported corresponding values of 8.4% unilateral and 2% bilateral posterior crossbites comparable with the current findings [3]. The prevalence of unilateral posterior crossbite is often associated with midline shift, which was found in 29% of the children. Males (32%) were more affected than females (26%). It is generally considered that the over-retained deciduous teeth, which rated 30 of the sample, is one aetiological factor of unilateral crossbite and the resulting midline shift, especially in males [29].

Deepbite that exceeded 4 mm was found in 28% of the sample. This was in accordance with the previous investigations in Morocco, Saudi Arabia and India [13, 28, 30], but it was higher than what has been reported in Turkish children and Iranian children, 18.3%, and 17.8% respectively [24, 26]. Most children at this age haven’t finished mandibular growth or have their second molars erupted yet; this may contribute to the increased overbite [1].

AOB was only noticed in 9% of the sample of the vertical anomalies, which is close to findings in studies conducted in Brazil [31], Colombia [22] and Saudi Arabia [13]. The current investigations indicated an increase in the prevalence of posterior openbite (16%). Many children at this age have an incomplete eruption of premolars; this may contribute to the high prevalence of posterior openbite.

Crowding was the most common alignment anomaly in the maxillary and mandibular arches, with a prevalence rate of 35% and 31%, respectively. Spacing was less than crowding and was present in 24% and 15% of the maxillary and mandibular arches, respectively. These results agree with other studies [4, 32]. Although spacing anomalies are not a disease in itself, they can act as a catalyst for other oral disorders, such as periodontal disease, caries, and temporomandibular dysfunctions, depending on the severity of the condition [33].

Males had more generalised spacing than females. This difference was statistically significant. The reason may be that about half of the children in the sample with maxillary spacing had diastema, and eruption of lateral incisors, and canines is slower in males than females, resulting in slower diastema closure [34].

Some limitations should be considered when comparing our results with other studies. Due to different methods and indices applied at varying age ranges among the population. Furthermore, neither impression models nor radiographs were used in this study. The possibility of under or overestimating from data collectors, the frequency of some criteria such as congenitally missing teeth, ectopic teeth eruption and the accuracy of space discrepancies analysis must be considered. Moreover, confounding factors may influence our results, especially in the occlusal examination.

Despite these limitations, this knowledge is essential to understand better Palestinian children’s malocclusion pattern and its relationship to demographic factors. It could be considered as a baseline for further diagnostic and preventive projects.

## Conclusion

Class I molar relationship is the most prevalent occlusal pattern among Palestinian schoolchildren. Different patterns of anterior openbite, anterior crossbite, midline shift, diastema, reduced overbite and overjet, spacing, and crowding exist in the West Bank of Palestine. Midline shift and diastema were reported more in males.

## Data Availability

The datasets used and analyzed during the current study are available from the corresponding author upon reasonable request.

## Abbreviations

OJ: Overjet
AOB: Anterior openbite
ICC: Intraclass correlation coefficient
WHO: World health organization
